# The Dark Side of Ultrasound Imaging in Parathyroid Disease

**DOI:** 10.3390/jcm12072487

**Published:** 2023-03-24

**Authors:** Roberta Centello, Franz Sesti, Tiziana Feola, Valentina Sada, Carla Pandozzi, Marco Di Serafino, Patrizia Pacini, Vito Cantisani, Elisa Giannetta, Maria Grazia Tarsitano

**Affiliations:** 1 Department of Experimental Medicine, Sapienza University of Rome, 00161 Rome, Italy; 2 Neuroendocrinology, Neuromed Institute, IRCCS, 86077 Pozzilli, Italy; 3 General and Emergency Radiology Department, “Antonio Cardarelli” Hospital, 80131 Naples, Italy; 4 Department of Medicine and Health Sciences “Vincenzo Tiberio”, University of Molise, 86100 Campobasso, Italy; 5 Department of Radiological Sciences, Oncological and Anatomo-Pathological Sciences, Sapienza University of Rome, 00161 Rome, Italy; 6 Department of Medical and Surgical Sciences, University Magna Graecia of Catanzaro, 88100 Catanzaro, Italy

**Keywords:** ultrasound, parathyroid, hyperparathyroidism

## Abstract

The diagnosis of parathyroid diseases by imaging still has some intrinsic technical limitations due to the differential diagnosis of different structures of the neck that mimic the parathyroid glands. In this view, ultrasound (US) is an established, low-cost, and non-invasive imaging technique that still represents the first-line approach for evaluating patients with parathyroid disease. The objective of this article is to provide a comprehensive review of the applications of USs in clinical practice, discussing the histopathological and US characteristics of the parathyroid glands in normal and pathological conditions, the advantages of preoperative imaging, and novel updates on the most useful and currently available multiparameter US techniques.

## 1. Introduction

Ultrasound (US) is considered the gold-standard diagnostic tool for neck organic disorders. In fact, the high-resolution and dynamic nature of this low-cost and non-invasive technique perfectly matches the superficial but complex anatomy of the neck, allowing the operator to obtain precise anatomic details and to identify pathological lesions in most cases [1].

Against this background, parathyroid disease still remains a diagnostic dilemma on US evaluation. In fact, whereas the clinical and biochemical diagnoses of altered parathyroid glands’ (PGs) function is in most cases straightforward, the radiological identification of the pathological glands, just like that of normal ones, can be arduous.

In case of hyperfunctioning PG(s), a clinical syndrome known as hyperparathyroidism (HPT) occurs, whose surgical-treatment planning is the main indication for performing imaging techniques to locate pathological parathyroid tissue.

As guidelines suggest, the aim of pre-operative parathyroid imaging is to achieve two concordant results from different imaging techniques. The combination of cervical US and nuclear imaging [i.e., ^99m^Technetium (^99m^Tc)-Sestamibi scintigraphy or single-photon emission computed tomography/computed tomography (SPECT/CT)] is acknowledged as the most widely used first-line strategy [2].

Cervical USs are performed with a high-frequency linear probe by transverse and longitudinal scanning of the neck, focusing on the paratracheal spaces and the carotid-jugular axis up to the carotid bifurcation and down to the sternal notch [3]. This technique presents clear technical advantages and good diagnostic accuracy, to the point that it has been recently advocated that it could be used as a main and sole investigation in the majority of patients [4]. Nonetheless, the US identification of parathyroid lesions can be difficult in many cases. This is mainly due to the morpho-pathological heterogeneity existing in the PGs disorders and to the numerous cervical lesions of other natures representing pitfalls to be aware of in the process of differential diagnosis. Moreover, an US’s ability to correctly identify enlarged PGs can be influenced and undermined by different factors, such as the presence of an ectopic PG below the VI neck level, a concomitant nodular thyroid disease, a prior anterior neck surgical procedure, or an unsuitable operator experience, which alone can account for as much as a 40% variation in the US sensitivity [2,5]. Yet in HPT affected patients, an accurate preoperative identification and localization of all hyperfunctioning glands by imaging methods is necessary not only for a more focused surgical approach (i.e., minimally invasive parathyroidectomy) with a subsequent reduction of surgery duration and complications compared with classical bilateral neck exploration, but also for long-term surgical success [6]. In these scenarios, nuclear-imaging investigations should be adopted. Besides the aforementioned traditional techniques, more recently positron emission tomography (PET)/CT using the radiotracer ^18^F-fluorocholine is emerging for its superior spatial resolution, lower radiation burden and shorter scanning time, and has been proposed as an alternative first-line “one-stop-shop” imaging method [7,8,9]. However, data on cost-effectiveness are currently lacking, and US remains the most useful available technique.

In recent years a multiparametric US approach has been adopted thanks to the introduction of some innovative problem-solving modalities which have further improved the ability of lesion characterization, offering greater diagnostic accuracy and leading to an increasingly important role of USs in clinical decision making. A multimodal US pathway includes, besides the traditional B-mode grey-scale and Color Doppler US, contrast-enhanced US (CEUS) and US-elastography (USE) [10,11,12].

CEUS is an approved, safe method consisting of the intravenous injection of a microbubble contrast agent during an US performance-detecting tissues characteristic microvascularization pattern [13,14,15]. The use of quantitative post-processing software tools, overcoming the limit of the subjective examiner’s interpretation, improves this technique’s diagnostic role, facilitating the differential diagnosis between parathyroid lesions and other mimicking structures with an accuracy of 96.4% [16,17].

USE refers to different dynamic technologies which non-invasively assess tissue elasticity through mild tissue deformation (strain USE) or sending focused acoustic impulses from modified transducers (shear-wave USE). Some authors demonstrated that USE may be a useful additional tool to improve the preoperative US detection of parathyroid lesions, as well as in discriminating between benign and malignant lesions [18,19,20]. 

However, to date there are no specific guidelines for the use of CEUS or USE for the evaluation of the PGs, and only sparse and incongruous literature data are available.

When, despite all of the available imaging methods, it results difficult to differentiate enlarged PGs from other lesions, US-guided fine-needle aspiration (FNA) cytology and the measurement of the FNA-parathyroid hormone (PTH) could be useful. As reviewed by Trimboli and colleagues, several studies demonstrated the relevance to measure FNA-PTH to localize parathyroid adenomas, reporting a sensitivity of 70–100% and a specificity of 75–100% [21]. However, this procedure is not well standardized, and no consensus exists about the FNA-PTH reference range [2]. Furthermore, it can be burdened with complications, such as post-FNA fibrosis (which makes surgery more difficult and may imitate malignancy on histopathological exam [22]), inflammatory reaction, parathyroid abscess, hematoma [23], parathyromatosis [24], and the potential risk of parathyroid carcinoma seeding [25]. Thus, FNA cytology is not widely recommended.

The aim of the current review is to present an overview of the histopathological and US characteristics of the PGs in normal and pathological conditions, discussing the clear advantages and the challenging aspects of traditional and novel multiparametric US techniques, with selected examples of clinical utility across a variety of lesions to consider for an adequate differential diagnosis.

## 2. Overview of Parathyroid Anatomy, Physiology, and Pathology

The PGs are endocrine glands responsible for producing PTH, whose principal role is to sustain or increase plasma calcium levels by acting directly on bones and kidneys and indirectly on the intestinal absorption through vitamin D action [26].

In normal conditions, the PGs measure 5 mm in length, 3 mm in anteroposterior diameter, and 1 mm in lateral diameter, and in most of cases four of them can be found: a superior pair typically located posterior to the mid-to-upper portion of the thyroid lobe, and an inferior pair generally found posterior to the lower thyroid pole [27].

However, a variability in the number and location of PGs has been reported. Supernumerary glands can be identified in 2–13% of the population, and because of their embryologic migration their location is variable; the superior glands can be ectopically located in a retropharyngeal or intrathyroidal site, while the inferior pair can be found from the angle of the mandible to the pericardium [28,29,30,31,32].

In general, normal PGs are barely identified on the US examination due to their intrinsic features or factors related to a poor sonographic window, such as in the case of thickened subcutaneous fat tissue and underlying thyroid disease [33]. In patients with HPT, the hyperfunctioning glands become detectable due to their size and echogenicity. 

Clinically, three types of HPT can be identified: primary HPT (PHPT), secondary HPT (SHPT), and tertiary HPT (THPT), corresponding from a morphological point of view to a spectrum of parathyroid lesions encompassing hyperplasia, adenoma (benign tumor), atypical tumors (tumor of uncertain malignant potential), and carcinoma [34]. 

PHPT is the result of an autonomous PTH oversecretion from abnormal PG(s) determining hypercalcemia, hypophosphatemia, and elevated urinary calcium, with potential complications on the skeletal, renal, neurocognitive, and cardiovascular systems [35].

Most PHPT cases occur sporadically, whereas about 5% are associated with a hereditary syndrome, i.e., types 1, 2A, and 4 multiple endocrine neoplasia (MEN) syndromes, HPT-jaw tumor syndrome, familial hypocalciuric hypercalcemia, neonatal severe HPT, and isolated familial HPT [36].

Overall, 80–85% of PHPT cases are caused by a single-gland adenoma [37].

Multiglandular parathyroid disease accounts for 15–20% of cases [37]. Traditionally, this clinicopathological entity has been referred to as “parathyroid hyperplasia”, but such a concept is no longer supported in the context of PHPT due to the fact that the affected glands are usually composed of multiple clonal neoplastic proliferations. Thus, to highlight its germline susceptibility-driven origin, the 2022 WHO Classification replaced the term of PHPT-related parathyroid hyperplasia with “multiglandular parathyroid disease” or “multiglandular parathyroid adenomas” [34]. Multiglandular disease is generally associated with inherited PHPT and should prompt consideration of genetic counseling and testing, as it could influence the management of affected patients [38].

The aforementioned new classification also replaced the term “atypical parathyroid adenoma” with “atypical parathyroid tumor” to indicate a parathyroid neoplasm of uncertain malignant potential, showing some histopathological findings typical of the parathyroid carcinoma (band-forming fibrosis, increased mitotic activity, presence of tumor cells within a thickened capsule) but lacking the definite diagnostic features of malignancy (invasion into adjacent tissues, vascular invasion and/or metastases) [34].

Rarely (<1% of cases) a parathyroid carcinoma is the PHPT’s underlying pathological lesion [37].

SHPT corresponds to a pathophysiological PTH oversecretion from hyperplastic PGs in response to a chronic stimulus that is usually a reduction in the serum calcium concentration due to an underlying chronic disorder (e.g., chronic renal failure, malabsorption syndromes, vitamin D deficiency). Moreover, drugs such as lithium and thiazide diuretics can be associated with increased PTH levels [39]. Whereas PHPT often manifests with a uniglandular disease, SHPT almost always manifests with multiglandular parathyroid disease so that in the current WHO Classification the term “parathyroid hyperplasia” is used primarily in the setting of secondary hyperplasia [34].

THPT reflects the result of a long-standing SHPT in which the stimulated PGs are no longer in a reactive mode but have assumed a quasi-autonomous function, not too dissimilar from PHPT, with the emergence of a PTH-producing adenoma or rarely carcinoma [39,40] and the development of refractory HPT and hypercalcemia, in patients with previously normal serum calcium levels [29,39]. In this condition, patients are consequently exposed to a potential risk of vascular and soft-tissue calcifications, and an adequate therapy may be indicated [41].

## 3. Ultrasonographic Findings of Parathyroid Glands

As mentioned above, normal PGs are scarcely detected among the structures of the anterior cervical region because of their small size, variable locations, and similar echogenicity to the thyroid gland or the perithyroidal fat tissue.

In normal conditions, in the rare cases where it is visible on the US examination, a PG appears as a very small oval/flat-shaped hypoechoic structure situated posterior to the thyroid lobes, between the trachea and the carotid artery [33,42]. Moreover, with advancing age, the PGs tend to accumulate more fat cells and granules, which probably makes them more echogenic and less conspicuous within the other echogenic adipose tissues of the neck [42].

**Figure 1:**
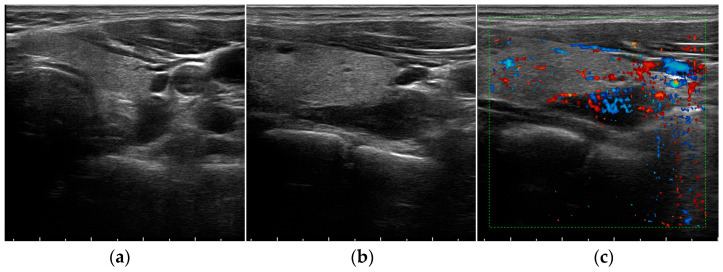

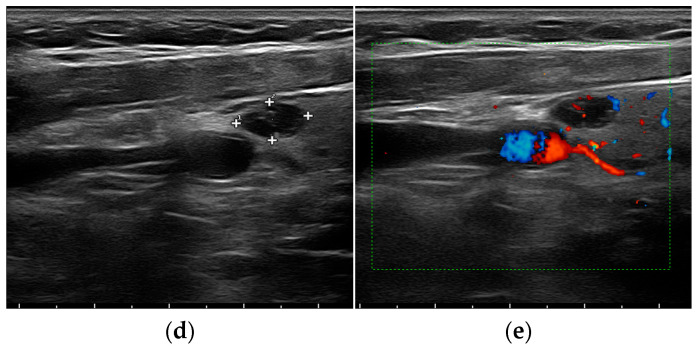
Grey-scale (**a**,**b**,**d**) and Color Doppler (**c**,**e**) aspect of a parathyroid adenoma.

**Figure 2:**
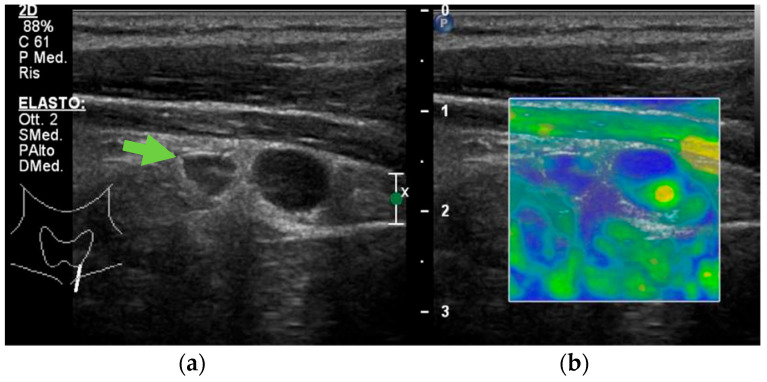
Grey-scale (**a**) and elastography (**b**) aspect of a hyperplastic parathyroid gland (arrow).

The usefulness of the US increases in pathological conditions [43]. In fact, hyperfunctioning PGs become more easily identified, usually appearing on the B-mode grey-scale US as enlarged, circumscribed, hypoechoic, oval-shaped lesions, delineated by hyperechoic connective tissue [17] (Figure 1a,b,d and Figure 2a).

The hypoechogenic aspect is due to the reduction of fat tissue components in these lesions [44]. However, they may have various shapes and sometimes present internal anechoic areas with dorsal echo amplification because of cystic degeneration following the retention of colloid secretion or after hemorrhage [45]. Cystic inclusions may be seen in both malignant and benign lesions, but are generally associated with hyperplasia more than adenomas [17].

It is virtually impossible to sonographically differentiate glandular hyperplasia from adenomatous formations. In case of multiglandular disease, more than one symmetrically or asymmetrically enlarged, lobulated gland may be seen [42].

On the Color Doppler US imaging, both adenoma and hyperplasia in most cases show prominent feeding polar vessels entering the pole and then extending around the periphery of the enlarged gland (Figure 1c,e) [46,47].

Similarly to all the other imaging methods, US is less sensitive for the detection of multiglandular- than of single-gland disease as hyperplastic PGs are usually significantly smaller [48].

A systematic review by Ruda et al. including more than 20,000 patients with PHPT reported sensitivities of 78.5%, 16.2%, and 34.9% for single-gland, two-gland, and multiglandular parathyroid disease, respectively [49]. However, compared with nuclear medicine imaging methods such as ^99m^Tc-Sestamibi dual-phase scintigraphy and SPECT/CT, the US still has been reported to have a slight advantage in the localization of hyperplastic PGs. The combination of US and scintigraphy or SPECT/CT could improve the accuracy in localization of parathyroid hyperplasia, and should be considered the first-line method in such cases [50]. 

As far as for the PHPT pluriglandular involvement, and also for SHPT cases, imaging is not always able to highlight all four glands, mainly due to the morphopathological heterogeneity existing in this disease. However, performing an US examination prior to surgery can be useful to obtain a suggestive cervical map and an evaluation of the thyroid gland and its possible associated lesions [51].

Currently, there are no well-defined US characteristics published in the literature that allow practitioners to distinguish a parathyroid carcinoma from a benign parathyroid tumor. Nonetheless, some US features may raise the suspicion for malignancy: >3 cm length, a depth/width ratio > 1, lobulated hypoechoic/heterogeneous aspect, irregular borders, thick capsule, suspicious vascularity, and intra-nodular calcifications [52,53]. However, these features may also be seen in benign tumors, and thus cannot be decisive for a preoperative diagnosis. Occasionally, the infiltration into surrounding tissues and cervical lymph node enlargement can be identified or suspected [26].

Of note, longstanding SHPT and THPT may be associated with enlarged PGs showing some atypical features that can mimic invasive growth. Thus, caution is urged before a preoperative suspect of parathyroid carcinoma is made in the setting of an advanced chronic renal failure [34].

On CEUS evaluation, parathyroid hyperplasia is characterized by fast intense homogeneous enhancement and a homogeneous wash-out appearance, whereas adenomas show an early peripheral hyperenhancement with central wash-out in the later phases [16,17,54].

To the best of our knowledge, only one case report where CEUS was performed on a parathyroid carcinoma has been published. In such a case, CEUS was highly suggestive of malignancy, with early heterogeneous enhancement and a homogeneous early wash-out [55].

Differences in the histopathological structures of normal and adenomatous PGs are responsible for the variations of gland stiffness detectable with USE. Indeed, because of their reduced fat-tissue components and external fibrous hard capsules, parathyroid adenomas display a significantly increased stiffness compared with normal and hyperplastic PGs, but a reduced stiffness when compared with parathyroid carcinoma (Figure 2b) [19,20].

The identification of an intrathyroidal parathyroid adenoma can be challenging. Characteristically it appears as a solid, profoundly hypoechoic nodule compared with other thyroid nodules or thyroid parenchyma, partially or fully enveloped with thyroid tissue, and a polar feeding vessel identified on Color Doppler US. The operator should be more suspicious of an intrathyroidal parathyroid adenoma especially in front of a patient with high clinical and biochemical probability of HPT when a nodule with these characteristics is noted and an extrathyroidal parathyroid adenoma is not visualized [56].

In cases of suspected ectopic PGs, the combination of the US with other radiological techniques, such as ^99m^Tc-Sestamibi scintigraphy and magnetic-resonance imaging (MRI), must be considered as this has a clear advantage in improving preoperative imaging accuracy, allowing for a minimally invasive surgical approach for patient’s treatment [50]. Table 1 summarizes the main US characteristics of parathyroid lesions.

## 4. Ultrasound Mimics of Parathyroid Glands

Several neck structures may show visual similarities with enlarged PGs so that to differentiate them on a conventional US can represent a compelling challenge for the clinician [57]. As reported by numerous authors, each neck structure presents characteristic vascularization patterns and elasticity [19,58]. According to this evidence, the multiparametric US turns out to be very useful in differentiating enlarged PGs from other lesions such as thyroid nodules, neck lymph nodes, or paragangliomas.

Conventional US sensitivity is particularly low when the parathyroid disease is associated with goiter, especially when thyroid nodules are located posteriorly [59]. The reported prevalence of PHPT in combination with thyroid pathologies ranges from 17% to 84%; in this case, the preoperative PGs localization is known to have lower sensitivity and specificity [60]. In fact, enlarged PGs next to the thyroid poles might be misinterpreted as a part of the thyroid tissue [61]. Benign thyroid nodules present fast-in and slow-out homogeneous, intense smooth-rim enhancement on CEUS evaluation. In cases of malignancy, heterogeneous peripheral hypoenhancement in combination with internal enhancement patterns and a slow wash-in and wash-out curve lower than in normal thyroid tissue can be observed [62]. As for the USE examination, due to the fact that the thyroid gland is mainly composed of colloid-filled follicles, its appearance is soft [63]. Benign thyroid nodules appear softer than malignant ones and parathyroid adenomas [64,65]. Malignant thyroid nodules tend to have a higher stiffness compared with parathyroid adenomas, but lower compared with parathyroid carcinoma [19,64].

**Figure 3:**
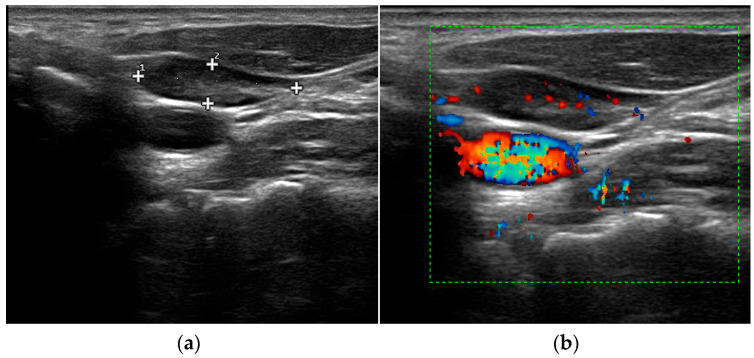
Grey-scale (**a**) and Color Doppler (**b**) aspect of cervical lymph node.

Perithyroidal lymph nodes, which are often identified in cases of chronic lymphocytic thyroiditis, may be factors leading to false-positive diagnoses of parathyroid incidentalomas [66]. In this context, the vascular pattern evaluation through Color Doppler US may be helpful for an appropriate differential diagnosis. Indeed, while PGs usually show polar feeding vessels, cervical lymph nodes characteristically present a hilar blood supply with feeding vessel entering the hilum centrally (Figure 3) [67].

However, these findings are not common in all parathyroid lesions and cervical lymph nodes [68]. Benign cervical lymph nodes generally display centrifugal and homogenous enhancement on CEUS evaluation. Conversely, according to their histopathologic changes, most metastatic/neoplastic lymph nodes are characterized by centripetal enhancement [69]. USE can be useful in distinguishing parathyroid lesions from cervical lymph nodes too, the former appearing significantly stiffer than the latter [70].

Paragangliomas are a rare form of slowly growing but potentially locally invasive, highly vascularized, neuroendocrine neoplasms arising from the chromaffin cells of sympathetic or parasympathetic paraganglia and accounting for the 0.6% of all head and neck tumors [71].

**Figure 4:**
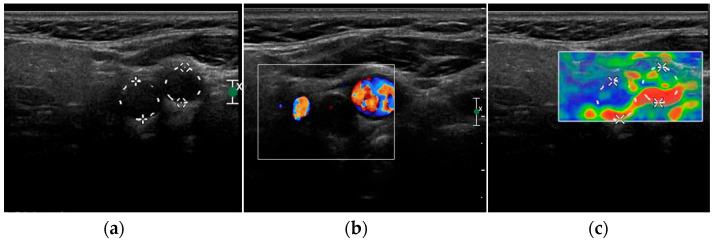
Grey-scale (**a**), Color Doppler (**b**) and elastography (**c**) aspect of a carotid body paraganglioma.

At the B-mode US, neck paragangliomas generally appear as well-defined hypoechoic masses that displace the carotid bifurcation, but can also show mixed echogenicity (Figure 4a) [14,72]. With Color Doppler imaging, an increased vascularity with a low-resistance flow pattern is generally evident, which can be predominantly peripheral with centripetal vessels inside the lesion [14]; however, a case of avascular paraganglioma is reported in literature [72] (Figure 4b). There is still limited evidence about CEUS appearance of neck paragangliomas [72,73]; these lesions normally show a strong and homogeneous contrast enhancement, such as that of the internal carotid artery, and a slow and progressive wash-out may be reported [14]. A clear and defined USE pattern has not been identified yet; the tumor can be both soft and hard [14] (Figure 4c).

**Figure 5:**
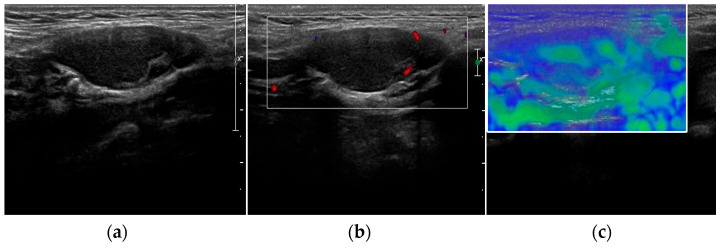
Grey-scale (**a**), Color Doppler (**b**) and elastography (**c**) aspect of a infected second branchial cleft cyst.

Branchial cleft cysts, benign lesions caused by the anomalous development of the branchial apparatus during embryogenesis, must be considered in the differential diagnosis. Anomalies of the second branchial cleft account for approximately 90% of all cases and sonographically typically present as round-oval, hypo- to anechoic masses with well-defined margins, thin walls, and posterior wall enhancement, which compress the surrounding soft tissues [74]. However, there is variability in their US appearance. In fact, in the presence of infections or abscesses, the content of the cyst may become corpuscular, resulting in a pseudo-solid heterogeneous appearance [75] (Figure 5a,b). No published data are available about their USE appearance, but in consideration of its histopathological features, it is presumable that they will present as soft as cysts of other nature (Figure 5c).

Hemangiomas are common benign vascular tumors characterized by the abnormal proliferation of endothelial cells and abnormal blood vessel structures which, unless thrombosed, have characteristic imaging features [76]. On the grey-scale US, hemangiomas usually appear as solid hyperechogenic masses, with central hypoechogenicity and echogenic capsule. At the Color Doppler US, they show a rich vascular density with a prominent high-velocity arterial flow [76]. In large hemangiomas, an US is usually combined with a cross-sectional MRI for further evaluations of neighboring vital structures, such as the upper aerodigestive tract and the cervical vessels [77].

## 5. Complementary Imaging Techniques

The identification of hyperfunctioning PGs is mandatory for an adequate treatment of HPT, and in most of cases a stepwise approach with US and subsequent ^99m^Tc-Sestamibi SPECT/CT is the first-line imaging strategy [2,78]. In cases of negative or inconclusive conventional imaging methods, ^18^F-fluorocholine PET/CT with its high spatial resolution, low radiation burden and short scanning time has been proposed as an alternative first-line “one-stop-shop” imaging method [7]; nevertheless, data on cost-effectiveness are currently lacking [2,78]. A further reduction of radial exposure can be obtained by the combination of ^18^F-fluorocholine PET and MRI [2,78]. Moreover, ^18^F-fluorocholine PET/MRI can be particularly useful in patients with low ^18^F-fluorocholine uptake [79], as well as in patients with discordant or inconclusive results of standard imaging methods [2,78]. However, ^18^F-fluorocholine PET/MRI-integrated systems are not widely available. Patients with a distorted cervical and chest anatomy or with small ectopical hyperfunctioning PGs may benefit from a four-dimensional (4D)-CT, which has a comparable sensitivity to ^99m^Tc-Sestamibi SPECT/CT, with the drawback of a relatively low specificity and a significant radiation burden [2,78]. A combination of ^18^F-fluorocholine PET and 4D-CT can be useful in complicated cases, being superior to ^18^F-fluorocholine PET and 4D-CT alone in the localization of the hyperfunctioning PG [80]. MRI alone can be a viable choice as a second-line imaging technique; however, this can be considered as a first-line strategy in pregnant women [2,78].

## 6. Conclusions

For most of other neck organic disorders, the US cervical examination is the first-choice imaging tool for HPT-affected patients. Regardless of HPT etiology, surgical treatment is the recommended management strategy in patients meeting the international guidelines criteria and with no contraindications [81]. Bilateral neck exploration has been a widely used method for many years, but in the last decade it has been increasingly replaced by the minimal invasive parathyroidectomy approach because of the latter’s smaller extent, shorter duration, and lower rates of complications. However, surgery efficacy strictly depends on the precise localization of the pathological parathyroid tissue using preoperative imaging techniques. Despite newer emerging radiological investigations, cervical US and scintigraphy remain the main methods used for this purpose [81].

There is no doubt that the preoperative US assessment of HPT patients has many advantages, such as low costs, high availability, no patient exposure to ionizing radiation, and good visualization of the neck morphology. In most situations its sensitivity and specificity match those of other imaging techniques, but it should be noted that there are some limitations too, as in cases of uncommon PGs features and locations and the presence of anterior cervical mimics that may represent pitfalls in the US differential diagnosis of parathyroid diseases.

In these cases, a multiparametric approach, including beside the conventional US and other imaging techniques such as CEUS and USE, can be helpful, and should be adopted to guide a proper work-up.

## Figures and Tables

**Table 1:** Ultrasound (US) features of parathyroid lesions on B-Mode, Color Doppler, contrast-enhanced US (CEUS), and US-elastography (USE) examination.

	B-Mode	Color Doppler US	CEUS	USE
Parathyroid hyperplasia	More than one symmetrically or asymmetrically enlarged, hypoechoic, oval shaped, lobulated gland. Significantly smaller than adenoma. Cystic inclusions may be seen.	Feeding polar vessels entering the pole and then extending around the periphery.	Fast intense homogeneous enhancement. Fast homogeneous wash-out.	Stiffer than proper parathyroid glands.
Parathyroid adenoma	Enlarged, circumscribed, hypoechoic, oval shaped lesion, delineated by hyperechoic halo. Cystic inclusions may be seen.	Feeding polar vessels entering the pole and then extending around the periphery.	Early peripheral hyperenhancement. Central wash-out in the later phases.	Stiffer than hyperplastic parathyroid glands.
Parathyroid carcinoma	Length > 3 cm, depth/width ratio > 1. Lobulated, heterogeneous, hypoechoic lesion. Irregular borders. Thick capsule. Intranodular calcifications. Cystic inclusions may be seen.	Intralesional disordered vascularity.	Early heterogeneous enhancement. Early homogeneous wash-out.	Stiffer than proper, hyperplastic, and adenomatous parathyroid glands.

## Data Availability

Not applicable.
